# New Approaches on Japanese Knotweed (*Fallopia japonica*) Bioactive Compounds and Their Potential of Pharmacological and Beekeeping Activities: Challenges and Future Directions

**DOI:** 10.3390/plants10122621

**Published:** 2021-11-29

**Authors:** Alexandra-Antonia Cucu, Gabriela-Maria Baci, Ştefan Dezsi, Mircea-Emil Nap, Florin Ioan Beteg, Victoriţa Bonta, Otilia Bobiş, Emilio Caprio, Daniel Severus Dezmirean

**Affiliations:** 1 Faculty of Animal Science and Biotechnology, University of Animal Sciences and Veterinary Medicine Cluj-Napoca, 400372 Cluj-Napoca, Romania; antonia.cucu@usamvcluj.ro (A.-A.C.); gabriela-maria.baci@usamvcluj.ro (G.-M.B.); victorita.bonta@usamvcluj.ro (V.B.); ddezmirean@usamvcluj.ro (D.S.D.); 2 Faculty of Geography, Babeş-Bolyai University, 400084 Cluj-Napoca, Romania; 3 Faculty of Geodesy, Technical University of Civil Engineering Bucharest, 020396 Bucharest, Romania; mircea.nap@usamvcluj.ro; 4 Faculty of Horticulture, University of Animal Sciences and Veterinary Medicine Cluj-Napoca, 400372 Cluj-Napoca, Romania; 5 Faculty of Veterinary Medicine, University of Animal Sciences and Veterinary Medicine Cluj-Napoca, 400372 Cluj-Napoca, Romania; florin.beteg@usamvcluj.ro; 6 Department of Agricultural Sciences, University of Naples “Federico II”, Via Università, Portici, 100-80055 Naples, Italy; emilio.caprio@unina.it

**Keywords:** *Fallopia japonica*, Japanese knotweed, antimicrobial activity, antioxidant effect, bioactive compounds, honey, invasive species, phytopharmaceuticals

## Abstract

Known especially for its negative ecological impact, *Fallopia japonica* (Japanese knotweed) is now considered one of the most invasive species. Nevertheless, its chemical composition has shown, beyond doubt, some high biological active compounds that can be a source of valuable pharmacological potential for the enhancement of human health. In this direction, resveratrol, emodin or polydatin, to name a few, have been extensively studied to demonstrate the beneficial effects on animals and humans. Thus, by taking into consideration the recent advances in the study of Japanese knotweed and its phytochemical constituents, the aim of this article is to provide an overview on the high therapeutic potential, underlining its antioxidant, antimicrobial, anti-inflammatory and anticancer effects, among the most important ones. Moreover, we describe some future directions for reducing the negative impact of *Fallopia japonica* by using the plant for its beekeeping properties in providing a distinct honey type that incorporates most of its bioactive compounds, with the same health-promoting properties.

## 1. Introduction

Plants have always played an important role in human life because of their nutritional and health benefits and are therefore considered to be nutraceuticals [1,2,3,4]. Their use as alternative medicine continues to remain popular, despite the progress of the pharmaceutical industry. Moreover, in light of the impact that food can have on human health [5], consumers have become more sensitive towards balance-supporting food that can improve and enhance health [6,7].

In this light, Japanese knotweed, known also as: *Fallopia japonica*, *Reynoutria japonica (R. japonica*), *Polygonum cuspidatum (P.cuspidatum)*, according to *Flora Europaea* [8], is well known to be used as part of alternative medicine, especially to prevent or to treat various disorders [9].

Native to Asia (China, Japan, Taiwan, North and South Korea) and North America, Japanese knotweed is an herbaceous perennial plant [10], from the *Polygonaceae* family [11]. It is found in sunny places, on the banks of rivers, or in pastures due to the presence of nitrogen from agricultural practices [12].

First introduced in Europe for ornamental purposes during the 19th century [13,14,15], Japanese knotweed has become one of the most invasive European alien species [16] because of its ability to expand in different types of habitats [17,18].

Possessing long-branches, thickened rhizomes and large ovate or elliptical blade leaves [10,19], this fast-growing invasive plant is distinguished by some specificities such as strong regeneration capacity [18], ample environmental resistance to various habitat conditions [20], high hybridization potential [21] and clonal and sexual reproduction [22]. Thus, it is tremendously difficult and costly to remove from invaded areas or to control their spread, as numerous studies have proven [23,24,25,26,27,28,29]. Moreover, it exhibits a menacing character towards native flora and ecosystems [30] because of the inhibition mechanism; its chemical composition manifests on local vegetation [31,32,33]. In this regard, alien plants in general, and the Japanese knotweed in particular, significantly affect the economy and ecosystem of the areas that they aggressively take over [15,34], and are therefore seen as a growing threat to global sustainability [35].

While Japanese knotweed can have a significant negative impact on its surrounding environment, including the endangerment and extinction of local species [33,36], effecting crop production [37], human health [38,39,40], forest regeneration [35,40] and urban and rural land management [41], this invasive plant also has several positive aspects. Reports in the literature show that Japanese knotweed can have a positive influence in terms of its use as food and fodder [39,42], medicine [10,43,44,45], fuel [46] and bioenergy [47,48], as well as an ecological indicator [49], insecticidal and fungicidal product [50,51,52], organic fertilizer [12] or carbon adsorbent [53]. These latest positive uses of *F. japonica* can act both as a preventive method for its invasiveness, as well as a pathfinder for further and innovative utilization of this troublesome plant species in the current circular economy.

It is well known that plants possess important sources of biologically active compounds that act as natural antioxidants, possessing nutritional, functional, as well as important health benefits [54]. It is also the case for Japanese knotweed and largely its rhizomes, whose phytochemical composition has indicated the presence of important amounts of anthraquinones (emodin, citreorosein, fallacinol, physcion, etc.), flavonoids (rutin, apigenin, quercetin, quercitrin, isoquercitrin, hyperoside, reynoutrin and kaempferol), and most important, stilbenes (resveratrol and polydatin), coumarins, lignans, essential oils along with other compounds [55,56,57,58,59].

Among these compounds, the most studied, because of their pharmacological values, are resveratrol, polydatin, emodin and physcion [60,61]. According to clinical trials, the four most studied bioactive compounds have revealed positive pharmacological effects, including antioxidant [61,62,63], antimicrobial [64,65,66], anti-inflammatory [67,68,69], neuroprotective [70,71,72] or anticancer activities [73,74,75,76], among others.

As current knowledge about the medicinal characteristics of Japanese knotweed are still incomplete, the aim of this review is to provide a deeper understanding of the pharmacological effects that the bioactive compounds of this plant possess and to draw attention to its possible use as an alternative medicine for specific diseases.

Furthermore, based on the therapeutic potential of the bioactive compounds found in different parts of Japanese knotweed, future directions can be taken into consideration, to both reduce its negative impact and offer new perspectives on the sustainable landscape management of knotweeds [77]. Thus, by creating new medical and economic value products, we can encourage the re-examination of the use of herbicides as the main eradication method for this invasive plant [78].

From this perspective, our last chapter will focus on the potential of Japanese knotweed flowers as a nectar source for bees and the valuable health-enhancing properties of the honey it can produce.

Consequently, to carry out this review, the authors researched literature using Japanese knotweed, *F. japonica*, *R. japonica* or *P. cuspidatum* as the main keywords, focusing especially on the papers that underlined its chemical composition and its potential use as a pharmaceutical, as well as the activities of its bioactive compounds. The denomination used by different authors was kept in our manuscript, due to the fact that we investigated the same plant, according to different botanical specialists. In an effort to obtain thorough information, the most accessed databases were Web of Science, ScienceDirect and Google Scholar, where resveratrol, emodin and polydatin have been carefully covered, as to achieve a comprehensive understanding of the holistic therapeutic effects that this invasive plant and its compounds can exert on an individual’s health, highlighting in vitro and in vivo studies and results that demonstrate its antioxidant, anti-inflammatory, neuroprotective and anticancer properties. Moreover, in order to undertake a comprehensive research, the selection was unlimited and included articles up until the submission of the study.

## 2. Phyto-Chemical Constituents and Identification Methods

**Figure 1:**
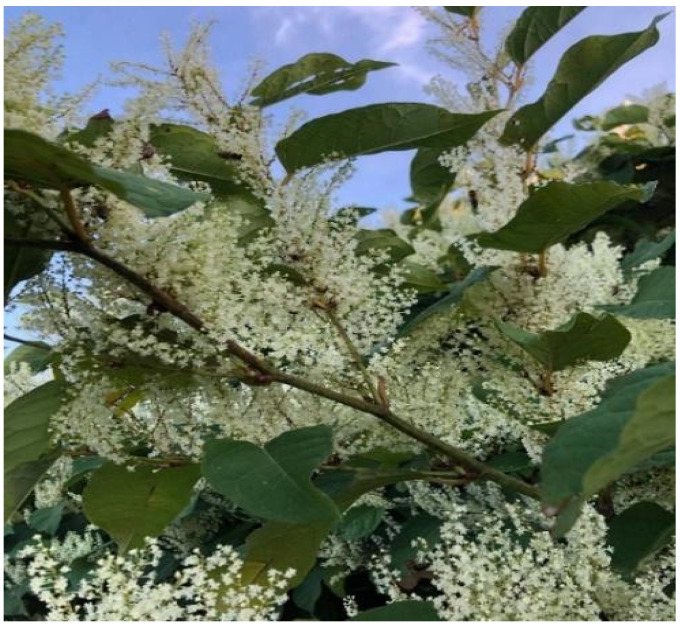
Japanese knotweed plant (Maris Marius Gabriel, beekeeper—personal collection).

As stated before, Japanese knotweed (Figure 1) has been studied for its chemical composition and properties.

Principal classes of compounds, considered as bioactive compounds, are flavonoids, stilbenes, anthraquinones, coumarins and lignans, found in roots, stems, leaves and flowers [10,57,58,79,80]. It is important to note that environmental factors, including climate or harvesting time, play a key role in the plant’s chemical composition [81]. Moreover, as a balance, Japanese knotweed can have a great impact in modifying the physico-chemical parameters of the soils they invade [82], so that it is able to adapt to any kind of soil and even enrich it with a lot of nutrients [83]. As a consequence, it is challenging to compare the differences between the phyto-chemical constituents, depending on the region from where the samples were collected.

In this regard, Table 1 presents the most representative studies made on the chemical composition of Japanese knotweed, emphasizing the part of the plant analysed, the phyto-chemical constituents found, the identification method, and some results.

Nawrot-Hadzik et al. (2018) revealed that the chemical composition of the roots of different species of *Fallopia (F. japonica, F. bohemica and F. sachalinensis)* contained a higher amount of piceid and resveratrol [57]. Comparing two *Fallopia* species, Lachowicz and Oszmianski (2019) reported that the leaves were a source of polymeric procyanidins, flavones, flavonols, phenolic acids, oleanolic and ursolic acids, while the roots contain flavan-3-ols and stilbenes, resveratrol being the most dominant [58]. Glavnik and Vovk (2020) extracted the anthraquinones from Japanese knotweed rhizomes using numerous extraction solvents and obtaining identical qualitative densitometric profiles in almost all the solvents with the exception of the sample test solution in dichloromethane, who allowed the extraction of physcion, emodin, and its equivalents (emodin-8-O-hexoside, emodin-O-acetyl-hexoside and emodin-O-malonyl-hexoside) [59]. Other compounds such as carotenoids were observed by Metličar et al. (2019) [80] when exploring the green and senescing leaves of two types of knotweeds, respectively, Japanese knotweed and Bohemian knotweed. Even if both plants indicated close pigment profiles, the total carotenoid content in green leaves exceeded the content revealed in senescing leaves for the two species, being comparable to one of the richest sources of carotenoids, specifically spinach [89,90]. Chen et al. (2013) have compared several Japanese knotweed samples from different parts of Canada and China, showing that the higher amount of resveratrol was found in the roots, compared to the stems and leaves. Moreover, it is suggested that the level of polydatin is similar in the samples from Prince Edward Island compared to those from China, while the level content of resveratrol turned up higher for the samples from China in contrast to those from Canada. In the case of emodin, Canadian samples contain a lower level than the Chinese samples, whereas physcion had higher levels in the Canadian samples compared to the Chinese ones [81]. However, Chu et al. (2005) founded in his study a higher amount of emodin, while physcion was found in the lowest quantity [84]. On the other hand, Vrchotová et al. (2007) evaluated the stilbene and catechin content of the spring sprouts and knotweed rhizomes of *Reynoutria species*, finding that the most predominant stilbene derivative in sprouts was piceid. By contrast, in the rhizome composition there was little resveratrol, while in the sprouts, the amount of resveratrol was much the same in all analysed knotweed samples [85]. Yi et al. (2020) managed to analyse five samples of the Japanese knotweed procured from different parts of China, reporting that from the seven compounds found, the greater proportion was represented by piceid [86]. It should be pointed out that according to the Chinese Pharmacopoeia, emodin and polydatin are considered to be standard indicators regarding the quality of dried material, thus a content of 1.0% emodin and 1.5% polydatin is required [19].

Studies made by Bensa et al. (2020) found that, concerning the degree of polymerization, leaves of the Japanese knotweed and other two knotweed species have similar chemical profiles of proanthocyanins [87]. This data is in accordance with the profiles of Japanese knotweed rhizomes examined by Glavnik et al. (2019) [91]. However, regarding the profile of gallates, leaves possess lower variety than rhizomes [87].

In his study, Alperth et al. (2021) detailed the presence of thirty-four substances, of which the greatest proportion was represented by anthranoids, while four new substances, namely demethylatedv torachrysonehexoside, sulfonyl torachryson, emodin methyl ether acetyl hexoside and malonylemodin, were declared new derivatives of the abovementioned compounds [88]. The aforementioned results are in agreement with the fact that the major chemical constituents found in the analysed parts of *F. japonica*, are a valuable source of polyphenolic compounds that exhibit a large spectrum of therapeutic properties and biological activities, which will be detailed as follows. As seen, the frequently used method for the identification of the biological constituents of this invasive alien species was chromatographic fingerprinting. This method is frequently employed for confirming the quality of plants used as alternative medical compounds or food supplements [87,92].

## 3. Biological Activities

In traditional medicine, *F. japonica* has been used for many years in Japan, China, Korea, and Taiwan. Recently, it became the spotlight of numerous studies, since its chemical composition has revealed promising therapeutic effects. Alcoholic, hydroalcoholic and water extracts from roots, rhizomes, and aerial parts (stems, leaves and flowers) have antibacterial activity [64,66,93,94,95], antioxidant effects [44,58,66,96,97,98,99,100,101,102,103,104,105], anticancer, antiproliferative and apoptotic properties [106,107,108], anti-inflammatory and antiviral activity [11,69,109,110,111,112] and many other bioactive properties.

The rich sources of biologically active substances found in *F. japonica* are essential for its specific therapeutic properties. Its applicability using in vitro and in vivo models has been largely investigated and is pointed out in the following section.

### 3.1. Antibacterial Activity

Recently, interest in antimicrobial substances originating from plants has extended to researchers investigating how different plants and their morphological extracts are associated with therapeutic activity and how they can be used as natural preservatives or ingredients for medicinal use. In this sense, *P. cuspidatum* exhibits significant antibacterial activities, mainly because of its bioactive compounds, especially stilbenes and anthraquinones. The most commonly used methods in analysing antimicrobial activity are disk diffusion, well diffusion and broth or agar dilution, together with the minimum inhibitory concentration (MIC) and minimum bactericidal value (MBC) [94]. Studies regarding antibacterial activity and bactericidal effects against different classes of microorganisms (Gram-positive and Gram-negative bacteria), including oral pathogens of *P. cuspidatum* extracts, were reported for several decades [93], without investigating the compounds, or classes of compounds responsible for this activity. Foodborne pathogens are represented by a large variety of microorganisms that lead to food spoilage and foodborne illnesses.

Lately, there has been an increase in interest, both from consumers and food producers, for safe food that is produced without using synthetic preservatives. Natural products with antimicrobial properties have been identified with plants as their main sources [64]. An excellent candidate for extracts with high antibacterial activity and, thus, important for food preservation is *P. cuspidatum.* A study by Shan et al. (2008), demonstrated that the major classes of constituents in the roots of *P. cuspidatum* are stilbenes and hydroxyanthraquinones [64]. The extract was tested on five foodborne pathogenic or faecal indicator bacteria, namely, *Bacillus cereus (B. cereus), Listeria monocytogenes (L. monocytogenes), Staphylococcus aureus (S. aureus), Escherichia coli* (*E. coli*) ATCC25922, and *Salmonella anatum (S. anatum)*. Results were reported using MIC, MBC, and observation of the bacterial morphology under scanning electron microscope of the incubated cells. Antibacterial results of the extract were verified using pure standards of the main compounds found in the root extract of *P. cuspidatum,* indicating high antibacterial activity for all of them. Except for *E. coli,* all other bacteria used in the study presented a high diameter of inhibition zone (DIZ) as average value compared to pure standards (6.4–20.2 mm compared to 15.2–35.4 mm). However, MIC and MBC were much better when compared to pure standards for Gram positive bacteria (*B. cereus, L. monocytogenes*), due to the synergism between compounds (156.3 µg/mL for *L. monocitogenes* compared to 312.5–625 µg/mL for pure standards and 312.5 µg/mL for *B. cereus* compared to 625 µg/mL for pure standards, excepting resveratrol with the same concentration as the extract). Another in vitro study demonstrated the potent inhibitory effect of *P. cuspidatum* and specifically of emodin against Gram-negative *Haemophilus parasuis* [113]. Therefore, the results indicated a MIC concentration of 32 g/mL and a MBC value of 64 g/mL for emodin.

Magacz et al. (2021) [95] proved the antibacterial effects of three *Reynoutria* species against *Streptococcus mutans (S. mutans)* and the positive impact on the prevention of caries formation. Due to the fact that extracts of the rhizomes of *Reynoutria species* are a rich source of polyphenols, they can balance the lactoperoxidase cycle, acting as activating/inhibiting agents for the antibacterial activity of the enzyme. The results showed that *R. japonica* extract exhibited the strongest inhibitory effect against the analysed microorganism using the acetone extracts (MIC 0.15 mg/mL), while for the bactericidal activity *R. Japonica* revealed a strong activity for the ethyl acetate fraction and acetone (MBC 0.60 mg/mL). Moreover, the authors observed that the content of polyphenols is a key factor for the presence of its antimicrobial activity, as the water fraction in the case of *R. japonica* revealed a very week inhibition against *S. mutans* (MIC 1.20 mg/mL) compared to the other studied fractions. On the same page, Nawrot-Hadzik et al. (2019a) [63] observed the dependence between the polyphenols and the antiseptic activity when testing the bacterial viability of the same three *Reynoutria* species (*R. Japonica, R. sachalinensis* and *R. bohemica)* against Gram positive bacteria (*Streptococcus mutans, Streptococcus salivarius, Streptococcus sanguinis, and Streptococcus pyogenes).* Compared to the other three species, *R. japonica* extract had the highest antibacterial activity against *S. mutans* (mean MIC 1000 μg/mL and MBC 2000 μg/mL), mainly because of the abundance of stilbene, aglycones and anthraquinone aglycone, respectively. The latest mentioned studies and their results indicate that *R. japonica* can be considered a useful antimicrobial agent for the profilaxy of dental pathogens.

### 3.2. Antioxidant Activity

The positive therapeutic effects of the major compounds found in *F. Japonica*, namely polyphenols, are associated with its antioxidant capacity and therefore can be related to the modulation of a range of receptors and metabolic pathways. [105]. Therefore, as inhibitors of free radicals, they may cause oxidative reactions in the human body and thus produce various disorders, especially cancer [96].

When evaluating antioxidant activity, several methods are used specific for different matrices. These in vitro methods have certain advantages, but on the other hand, they also exhibit several disadvantages and limitations when used on plants, foods, or different extracts. The most used methods are: 2,2′-azinobis-(3-ethylbenzothiazoline-6-sulphonate) scavenging activity, (ABTS+) 1,1-diphenyl-2-picrylhydrazyl (DPPH·) radical scavenging activity, Fe^3+−^Fe^2+^ transformation assay, ferric reducing antioxidant power (FRAP) assay, cupric ions (Cu^2+^) reducing power assay (Cuprac), Folin–Ciocalteu reducing capacity (FCR assay), peroxyl radical (ROO·), superoxide radical anion (O_2_·-), hydrogen peroxide (H_2_O_2_) scavenging assay, hydroxyl radical (OH·) scavenging assay, singlet oxygen (1O_2_) quenching assay, nitric oxide radical (NO·), oxygen radical absorbance capacity assay (ORAC), to mention the most important.

Considerable studies regarding the antioxidant activity of *F. Japonica* have been carried out worldwide. For instance, Lachowicz and Oszmiańsk (2019) [58] compared the antioxidative activity between *F. japonica* and *F. sachalinensis* plants and their morphological parts (leaves, stalks, and roots), noting that the average values in *F. japonica* were higher than in *F. sachalinensis*. As for the morphological parts, the lowest level of antioxidative activity was found in the stalks of both *Fallopia* sp. samples, measuring an average of 31.79 for the ABTS assay and 14.11 mmol Trolox/100 g DW for the ORAC assay, respectively. Still, both the leaves of *F. japonica* and the roots of *F. sachalinensis* showed the highest antioxidative activity (81.12/71.22-ABTS assay, compared to 30.42/30.85—ORAC assay- mmol Trolox/100 g DW). This suggests that the antioxidative capacity of plants can be attributed to the polyphenolic content of its compounds. Another study showed strong antioxidant activity for 32 compounds from *P. cupidatum* samples collected in China [114], specifically 92.7% inhibition at a concentration of 64 μg·mL^−1^.

Studies have proven the high correlation between the phenolic content and the antioxidant capacity in *P. cupidatum* species. For instance, Lazurca et al. (2012) analysed the phenolic content and antioxidative activity of *P. cuspidatum* buds from Romania in different solvents. The analysis revealed that the total phenolic content (TPC) ranged from 1048 ± 13 mg GAE/100 g to 1426 ± 15 mg GAE/100 g fresh buds. The results are in agreement with the antioxidant capacity, which varied from 89.034 to 182.483 μM trolox/g fresh weight buds, using an extraction index of 96.3. This demonstrates a positive correlation between the antioxidant activities and the total polyphenol content, but not necessarily strong enough (r = 0.5274, *p* < 0.01) [98].

On the other hand, Ardelean et al. (2016) [44] observed by measuring the root extracts of this plant that the phenolic content was 146.34 ± 5.36, expressed as milligrams of gallic acid equivalents (GAE)/g extract. This result exhibited a notable correlation with its antioxidant effect, namely 92%, by using a concentration of 1 mg/mL in ethanol 50%. Other studies have also explored the phenolic and antioxidant activity of *P. cupidatum*, showing higher results, while Chen et al. (2010) identified for the ethanolic extract from *P. cuspidatum* roots a phenolic content of 276.78 ± 39.31 mg/mL and 231.73 ± 5.04 mg/mL expressed as gallic acid equivalents [102], Hsu et al. (2007) remarked that the phenolic and flavonoid total contents in *P. cuspidatum* have much higher inhibitory activity against the DPPH radical (641.1 ± 42.6 mg/g and 62.3 ± 6 mg/g). This latest study proved also an important antioxidative effect (the half maximal inhibitory concentration—IC_50_ value of *P. cuspidatum* extracts represents 110 μg/mL in free radical scavenging assays, 3.2 μg/mL in superoxide radical scavenging assays, and 8 μg/mL in lipid peroxidation assays, respectively) [100].

Among all polyphenols, the one most frequently associated with the antioxidative effect are stilbenes [115], mostly resveratrol [99,103].

Still, there are recent studies which demonstrate that stilbenes may not be the only contributing factors to the antioxidative activity of Japanese knotweed, and that different compounds may also play an important role. In this regard, Lee et al. (2011) has pointed out no correlation between the antioxidative property and resveratrol or emodin [116], as the smallest extracts of *R. japonica* showed the higher antioxidant activity.

In addition, Pan et al. (2007) revealed that resveratrol extract from *P. cuspidatum* roots had a lower antioxidant activity than ethanol extract [101]. Additionally, recent contributions confirmed the presence of polydatin and epicatechin in higher proportion than trans-resveratrol [117,118] showing at the same time a stronger antioxidant activity than the previously mentioned compound (higher IC_50_; 7.08 g/mL or 31.02 M) [97].

Moreover, an investigation by Lachowicz et al. (2019) indicated that the greater radical scavenging capacity of the compounds found in two *Reynoutria* sp. extracts (*R. japonica* and *R. sachalinensis*) was given by catechins, procyanidin, trans-piceid and trans-resveratrolside (procyanidins derivatives) [58]. The same direction is confirmed by Nawrot-Hadzik et al. (2019a) who suggested that procyanidins can also be taken into consideration when speaking about the antioxidant potential of the the Japanese knotweed [63].

These data suggest that Japanese knotweed is a rich source of natural antioxidants that can be used in human diet as an ingredient in different foods or drugs in order to protect and prevent chronic diseases (cardiovascular diseases, cancer, diabetes, obesity, etc.).

### 3.3. Anticancer, Antiproliferative and Apoptotic Activity

The continuous research on cancer prevention and treatment has led to some notable studies that have revealed the chemopreventive role of the *Polygonum genus*, notably *P. cuspidatum*. As cancer is the result of some biochemical processes that produce oxidative damage and possible death to the cells, the phenolic and metabolite compounds found in the roots of this alien species plant have been screened in order to demonstrate their antiproliferative and apoptotic activities. The study carried out by Xie et al. (2014) [108] revealed that one of the active constituents of *P. cuspidatum*, namely resveratrol, had remarkable inhibition effects on three human hepatoma cell lines (HepG2, SMMC–7721 and BEL-7402), using concentrations ranging from 2.5 to 40 μM in a time-dependent manner. Among the tested cell lines, SMMC-7721 was the most sensitive to the resveratrol action. Moreover, the study also confirmed that resveratrol could prevent the apoptosis of caspase 3 and caspase 9, the main cells involved in this process. The antitumor effects of this compound were also proven in vivo, as doses of 10 and 30 mg/kg of resveratrol could significantly reduce tumour growth (41.7 ± 12.45% and 60.9 ± 9.9%, respectively). The extracts of the same plant, and once again resveratrol as the main compound, proved its efficiency in inhibiting the human oral cancer cells in a study carried out by Wang et al. (2019). Thus, the cisplatin-resistant cancer cells originated from a human tongue cancer cell line CAL 27 and were exposed to 150 μg/mL *P. cuspidatum* extract for different hours. The results have shown that for 48 h, the half maximal inhibitory concentration of the plant extract in the tested cancer cells was 110.34 ± 8.21 μg/mL [118]. Other antiproliferative effects of *P. cuspidatum* roots against human hepatocellular carcinoma cells of HepG2 and SMMC-7721 were investigated by Jiao et al. (2018) who observed that besides the cell apoptosis effects, polydatin could inhibit cell proliferation, invasion, and migration [106]. Furthermore, other research confirmed the chemopreventive properties of the juglone analogue 2-ethoxystypandrone isolated from the *P. cuspidatum* roots [107]. This compound is considered to be an inhibitor of the signal transducer and activator of transcription 3 (STAT3), an important constituent of hepatocellular carcinoma. Therefore, the study made by Li et al. (2019) demonstrated that 2-Ethoxystypandrone was able to block the STAT3 activation (7.75 ± 0.18 μM, using 50% concentration) to induce the death of hepatocellular carcinoma and to inhibit the proliferation of cancer cells (IC_50_ = 2.70 ± 0.28 μM) [107].

These dates are consistent with the idea that the extracts of the Japanese knotweed have shown promising healing effects on various types of cancer by inhibiting the growth and progression of cancer gene cells in different stages and in a time/dose-dependent manner.

### 3.4. Anti-Inflammatory and Antiviral Activity

Some bioactive compounds from the extracts of Japanese knotweed exhibit anti-inflammatory and antiviral activities. The anti-inflammatory and protective activities of *P. cuspidatum* on Dry Eye Disease (DED) was investigated by Park et al. (2018) with both in vitro and in vivo analysis. It was revealed that the active substances from the examined plant are represented by caftaric acid, rutin, quercitrin, resveratrol and polydatin, the latest being the crucial one. In vivo, these compounds had increased the tear volumes of the groups treated (using 100 and 250 mg/kg extract) to 5.95 ± 0.71 and 6.49 ± 0.89 mm (*p* < 0.05), respectively, in contrast to the exorbital lacrimal gland-excised group (4.33 ± 1.28 mm, *p* < 0.0001), whereas in vitro it had positive effects on inhibiting the hyperosmolar stress-induced inflammation by reducing the expression of COX-2,Bax, nuclear translocation of NF-B through the stimulation of PARP, NF-B and caspase 3 [11].

Furthermore, Yu et al. (2020) gave evidence that resveratrol, emodin-1-O-d-glucoside, and emodin-8-O-d-glucoside play crucial roles in the anti-inflammatory properties of *P. cuspidatum* [69]. In addition, the study revealed that different concentrations of resveratrol can have a significant contribution in modulating the inflammatory process, namely at a concentration of 100 M resveratrol mitigates the TNF- level with the inhibition rate of 51.55%, while half of the same concentration diminished the pro-inflammatory cytokines production levels of IL-6 and MCP-1 cells by 63.86% and 69.88% [69]. Besides, Hodgin et al. (2021) revealed that resveratrol extract from *P. cuspidatum* had a major contribution in attenuating the symptoms of the Gulf War Illness, associated in general with an inflammatory process. Results showed that by taking a dose of both 200 mg/day or 600 mg/day, the symptoms associated with this illness were diminished. On the contrary, the other bioactive compounds analysed from another plant (luteolin and fisetin) showed no significant effects [111].

As for the antiviral effects, several studies confirmed that the bioactive compounds extracted from the Japanese knotweed rhizomes possess high curative properties against viral microorganisms. For example, Liu et al. (2013) [109] underlined the positive effect that the roots and rhizomes of *P. cuspidatum* has on treating the Coxsackievirus B4 (CVB4) infection. Specifically, emodin was explored through in vivo and in vitro assays, suggesting that it can lessen both the replication of Hep-2 and CVB4 cells. Emodin together with ethyl acetate subfraction F3 from *P. cuspidatum* were also indicated to slow down the Epstein–Barr virus (EBV) lytic cycle in a dose-dependent manner (5.94 μg/mL and 4.83 μg/mL, respectively, at a half maximal effective concentration [110].

Zhang et al. (2015) examined the cytotoxicity of both resveratrol and polydatin on Enterovirus 71 (EV71) infected cells, suggesting that while resveratrol has inhibition activity against the production of inflammatory cytokines of EV71 (IC50 of 21.36 μg/mL at a concentration of ≤40 μg/mL), polydatin revealed a lower antiviral activity (IC50 of 979.66 μg/mL, at a concentration of 300 μg/mL) [119].

The report made by Nawrot-Hadzik et al. (2021) showed significant effects of the butanol fractions from *R. japonica* and *R. sachalinensis* (especially vanicosides) on the inhibition of SARS-CoV-2 Mpro cells (7.877 g/mL, 4.031 g/mL, respectively at IC_50_). However, these constituents may act in synergy with other compounds such as polymerized procyanidins that can also exhibit a strong inhibitory activity on the antiviral pathway, as suggested by previous authors [112].

### 3.5. Other Bioactive Properties

In addition to the aforementioned biological activities, Japanese knotweed possesses some other notable therapeutic activities. For instance, its cardioprotective effects were mentioned in studies focusing on hyperlipidaemia [120], ventricular remodelling [121], ischemia-reperfusion injury [122], myocardial infarction [123], where different compounds of *P. cuspidatum* (resveratrol and polydatin) were proven to improve the functions and mechanisms of some enzymes responsible for the good functioning of the heart. Moreover, promising results with regard to the neuroprotective effects of this plant were reported in animal models. Resveratrol exhibited positive effects on ameliorating anxiety [124] and alleviating depression activity by decreasing the malonaldehyde marker for oxidative stress, using 15 mg/kg/day extract [125]. Importantly, it has also been implicated in inhibiting the progression of Alzheimer Disease through the multiplication of neurons [126]. In addition, Liu et al. (2016) found out that resveratrol can reduce depression symptoms such as sucrose preference, lipid peroxidation, superoxide dismutase, immobility time and locomotor activity [127]. The same results have been confirmed by Wang et al. (2016) [114] and Moore et al. (2018) [128]. Polydatin [72], emodin-8-O-*β*-d-glucoside [70] and 2-methoxy-6-acetyl-7-methyljuglone [129], have also shown impressive results in improving the biological parameters for cognitive deficits or neuromuscular coordination.

The anti-diabetic activity of *F. Japonica* was demonstrated by its ability to scavenger the methylglyoxal reactions, responsible for the apparition of this chronic disease [130]. Several studies in this direction suggested its protective effects regarding diabetes by ensuring better glycaemic control [117,131,132,133,134]. Moreover, the extract of *P. cuspidatum* was described to be beneficial for gastrointestinal disorders, notably ulcers [135], as well as in respiratory dysfunctionalities [136] or obesity [121,137,138].

Subsequently, the positive effects on reproductive system disorders were outlined for some compounds found in the roots of Japanese knotweed. In particular, convincing evidence indicates that this invasive alien plant may be an ideal remedy for hormone replacement [139] through the enhancement of the oestrogen-sensitive cells.

All the described bioactive properties of *P. cuspidatum* confirm, once again, that this plant can be a valuable nutraceutical source for human health.

However, besides the positive activities of the biological active compounds of *F. japonica*, we cannot ignore the possible toxicity that may occur. In this regard, resveratrol has been revealed to exert undesirable effects depending on the dosage intake, the most common being abdominal discomfort, diarrhoea and nausea [140,141,142,143], while emodin inhibited human sperm functions [144]. Therefore, it is important to underline that further investigations and clinical trials are required in order to establish quantitative assessment of the standardized dose that can be used in order to maintain the health contribution of this compound and to avoid side-effects.

## 4. Future Directions

### 4.1. Fallopia japonica Flowers: Important Nectar Source

It is worth mentioning that *F. japonica* is one of the invasive plants that are considered to produce pollination services [145]. Even if the male flowers are very rare for pollen production, its female flowers are a great source of nectar rich fructose and glucose and, therefore, are intensively visited by pollination-insects, respectively bees [18,146,147,148].

To better appreciate its importance for beekeeping, but also to determine the massive unwanted invasion, different modern techniques may be used to detect the presence of Japanese knotweed. The newest and, at the same time, the most efficient of these methods, both in terms of actual working time in the field and of the surfaces covered in a certain time interval, is the technique that uses U.A.V. (Unmanned Aerial Vehicle) technology, which, depending on the chosen device model and the sensors mounted on it, can cover areas between 10 ha and 100 ha in less than two hours of flight [149]. During a photogrammetric operation, all data will be recorded in a series of three-dimensional coordinates, in a graphical form. From the existing varieties of this type of technology, drones that successfully combine all the good elements from each major category, in a single device, seem to be at present, the one best choice, for almost any kind of large surface work: this being about Fixed Wing Vertical Take-Off and Landing U.A.V. Some of the most relevant software for capturing or processing photogrammetric data are DroneDeploy, Agisoft Photoscan, and Pix4D. Referring to the correct choice of sensors to be used in this field, multispectral cameras are by far the most useful accessories. With their help, different types of vegetation can be identified and differentiated (either cultivated or wild), and it is also possible to study and establish their condition [149]. This method may be used successfully for the mentioned purposes.

It is known that the beekeeping value of *F. japonica* originates in the Far East, but as the plant became naturalized in Europe and America, this property was studied in the respective areas also. The study of Ferrazzi and Marletto (1990) is one of the few published investigating the beekeeping potential of the plant [146]. Conducted in the Piedmont region of Italy between 1988 and 1989, the study describes the feeding behaviour of the bees during the flowering period from July to October. The tiny greenish-white flowers, placed in axillary spikes, produce a high amount of nectar from non-differentiated nectar tissue located at the base of the stamens. The flower has eight stamens, presented regularly shaped anthers, in which however no pollen was ever observed. One of the few studies on nectar of *P. cuspidatum* was made in Poland in 2001 [150], where in three consecutive years (1998–2000), different parameters were measured, during the blooming period which was determined between 01.09 and 23.09. This period is also in accordance with different observations made by beekeepers in Romania. As stated by the previous study, *P. cuspidatum* (*F. japonica*) has a good potential for honey production, as the nectar of *Polygonum* flowers owns a concentration of sugars ranging from 30 to 56%, and a potential for honey production of 200–355 kg/ha, with a daily gain of honey production of 10–15 kg [150]. Still, the nutritional quality of the invasive *F. Japonica* nectar, compared with other invasive plant species, is unknown.

Foregoing descriptions of the plant demonstrate a very good source of secondary metabolites from different chemical classes, from root to the flowers. Nectary glands also contain high amounts of bioactive compounds from the class of polyphenols, which are transferred to honey when the bees are collecting the nectar.

On account of this, the management control and eradication of *F. japonica* has to be made using sustainable methods that will keep creating a healthy environment and protection for biodiversity [151]. Thereby, the effects that some methods may have on insect communities found in invaded locations need to be consciously evaluated [152].

### 4.2. Knotweed Honey

**Figure 2:**
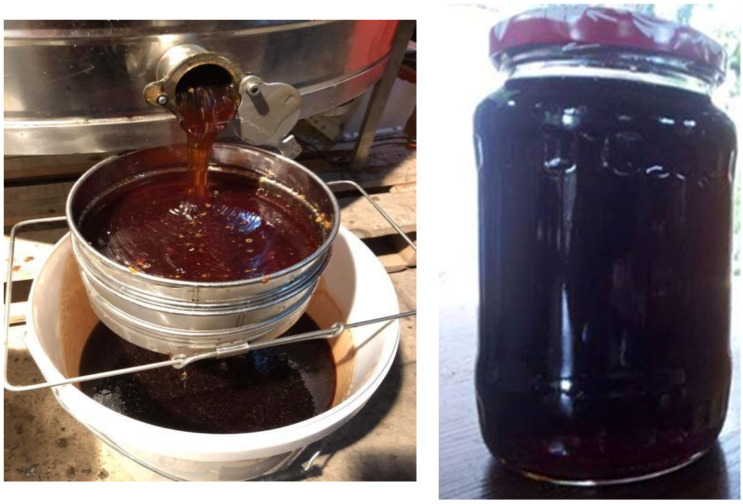
Colour of Japanese knotweed honey (Maris Marius Gabriel, beekeeper—personal collection).

As known, honey is the only sweet natural product, made by the bees from flower nectar or from sweet secretions of plants or found on the plants, brought to the hive and left for ripening and maturation [153]. Depending on the nectar source, honey is classified as monofloral, multifloral or honeydew honey. Its aspect, taste, and chemical composition, depends also on the same parameters. Another important factor for honey colour is the harvest period. For instance, honey collected in spring and early summer is light in colour, whereas in mid-summer, the colour of honey transforms to dark yellow, orange or even brown. The darker the honey colour is, the higher its nutritional value, represented by vitamins, minerals and secondary metabolites coming from the plants which provide the sugar source. Japanese knotweed plants belong to the *Polygonaceae* family, from which dark coloured honeys are produced (e.g., buckwheat honey). Japanese knotweed honey makes no exception. It was known and consumed in Japan for many years, from where the plant is native, but its invasiveness in countless regions of the world made this honey known, analysed, and appreciated. Knotweed honey is dark in colour (Figure 2), very aromatic, with a particular flavour, and it is finely and uniformly crystallized during storage [146]. In addition, it has an extremely high content of minerals, but also possesses antioxidant and antibacterial properties [154].

It is also known as “bamboo honey” due to the fact that the knotweed plant looks similar to a bamboo stick. Describing the chemical composition of Italian knotweed, Ferrazzi and Marletto (1990) stated that it has reduced humidity (17.9%) and medium electrical conductivity value, although it is very dark in colour (0.432 mS/cm), acidic pH (3.8) and has low acidity (free acidity 13 meq/kg, lactone acidity 1.25 meq/kg) and a small value of ash content (0.09%) [146].

A recent study [149] found similar characteristics for knotweed honey from western Romania. Due to the fact that this type of honey is harvested very late (generally October), there are few beekeepers in Romania producing it. Three samples were harvested in different locations from western Romania and analysed for their chemical composition, showing an average water content (15.6–20.0%) and electrical conductivity characteristic to multifloral honey (0.541–0.697 mS/cm), high diastazic index (11.52–18.74 DN) and low HMF content (0.29–9.93 mg/kg). Fructose and glucose are the main sugars found in knotweed honey, having similar values (38.9–39.8% fructose and 29.3–35.2% glucose). Therefore, the crystallization process installs very quickly from the time of harvesting. Other sugars identified were sucrose, turanose, maltose, trehalose and erlose (values from 0.13 to 3.18%). An important characteristic for this type of honey is total lipid and total nitrogen contents. High values (for honey) were quantified, ranging from 0.12 to 0.52% (total lipids) and 0.47–1.05% (total nitrogen). Further studies are necessary for the identification of amino acids and fatty acids, important compounds of this type of honey.

High amounts of minerals (K, Ca, Na and Mg) were also quantified in knotweed honey (6196.8 mg/kg for K). This amount is extremely high compared to other floral honeys, including buckwheat and manuka honey [155,156,157,158]. The polyphenolic content of honey came from the plants that ensure the nectar and pollen as raw materials for the bees. As stated before, Japanese knotweed is an important source of secondary metabolites from the classes of polyphenols, specifically stilbenes and anthraquinones. Total phenolic content of knotweed honey ranged between 100 and 195 mgGAE/100 g honey and 20–55 mgQE/100 g content of flavone/flavonols [154]. These values were higher compared to other dark coloured honeys, including buckwheat honey [159,160,161,162].

### 4.3. Comparison with Other Honey Types from the Same Plant Family

*F. japonica* (*R. japonica, P. cuspidatum*) belongs to the *Polygonaceae* family. According to Sanchez et al. (2009) [163] and Burke et al. (2010) [164], the phylogenetic tree for the *Fallopia, Reynoutria* and *Polygonum* is: *Polygonaceae, Polygonaceae, Polygonum, Fallopia* and *Reynoutria*. Searching for studies describing the chemical composition of Japanese knotweed honey, we could not find extensive literature, although this type of honey has been produced and consumed for many years, first in Japan, where the plant is native and later, all over the world where it has spread and maintained its common use. The few studies were compared to other dark coloured honeys, including buckwheat (from the same plant family) or manuka honey.

Manuka honey is known for its medicinal value more than for its taste, which is not appreciated by many consumers. It has a dark colour when fluid and freshly harvested, and its colour intensity decreases when crystallization occurs [155,165]. The smell is described as “pungent” most of the time and is not consumed for its aroma. Although, the health properties of dark honeys are by far superior to light colour honeys. From the chemical composition of honey, different classes of compounds found in very small amounts, determine their bioactive properties. The synergism between phenolics, organic acids, enzymes or peptides seems to be responsible for these properties. In the case of manuka honey, its bioactive properties are related to Unique Manuka Factor (UMF), representing the content of methylglyoxal and phenolic content. According to different studies [155,166,167,168,169], manuka honey contains high amounts of caffeic, p-coumaric, syringic acids, quercetin, luteolin, pinocembrin, isorhamnetin, kaempferol, chrysin, galangin, pinobanksin, methyl syringate, leptosin, glyoxal, and methyl glyoxal. The synergism between these classes of compounds present high antioxidant and antibacterial activity [170].

Buckwheat honey is produced in North America, but also in Europe, China and Russia. The main producers in Europe are Poland, Ukraine, the Netherlands, Germany and Moldova. This honey is characterised by a dark purple colour, almost black, with a malty aroma and strong animal odour [171]. Many scientific studies have shown the high antioxidant and antibacterial properties of this honey [172], especially its efficacy in respiratory tract infection. The study by Pasini et al. (2013) [171] highlighted the presence of p-hydroxybenzoic, p-coumaric, ferulic, syringic and caffeic acid, also abscisic acid, and quercetin, apigenin, pinobanksin, kaempferol, isorhamnetin, chrysin, pinocembrin and galangin in buckwheat honey. For this reason, the bioactive properties of this honey type are extremely high.

Furthermore, a study by Dzugan et al. (2020) [173], demonstrated that Polish buckwheat honey possesses the strongest antibacterial and antioxidant properties, because it is registered as the darkest Polish honey and, according to other scientific studies, the darker the honey, the higher the bioactive properties. Earlier in 2018, Deng et al. [157] comparatively evaluated the cellular antioxidant activity of buckwheat and manuka honey samples, using a cell-based model of HepG2 cells. The cellular antioxidant activity can determine the antioxidant activity more accurately than chemical-based methods, due to the fact that it takes into consideration the bioavailability, distribution of bioactive compounds in the interested biologic system and the cellular uptake. The EC_50_ value of buckwheat honey was lower than of manuka honey, indicating a potent cellular antioxidant activity for buckwheat honey.

Unpublished studies by Bobis et al. (2018) [174], presented at the Symposium Apimedica and Apiquality (Sibiu, Romania), highlighted the bioactive properties of knotweed honey (*F. japonica*), identified using HPLC, p-OH-hydroxybenzoic acid, syringic acid, p-coumaric acid, ferulic and t-cinnamic acid in honey samples from Romania. Additionally, a high percentage of inhibition of the DPPH radical was determined (61.69–71.75%), higher than registered for honeydew honey. Regarding the antibacterial activity, Japanese knotweed honey from Romania inhibits the growth of *S. aureus*, and is effective also on *Staphyloccocus pseudointermedius*, *Bacillus cereus* and *Salmonella enteritidis*.

Further studies of Japanese knotweed honey samples produced in different locations are needed to determine its chemical composition, biological properties and its best valorization.

## 5. Conclusions

Japanese knotweed, an all invasive plant species, represents a challenge because of its ecological and economic impact. Nevertheless, its chemical composition (from roots to leaves) has revealed the presence of unique active compounds that are reputable for their extensive variety of physicochemical features and biological activities. The aim of this review was to provide a comprehensive overview on the high therapeutic potential of Japanese knotweed by emphasizing its main compounds and their biological activities. Specifically, in vivo and in vitro studies on the chemical composition of *F. japonica* were covered, identifying resveratrol, emodin and polydatin as the main compounds that can exert therapeutic effects (antibacterial, antioxidant, anti-inflammatory and anticancer effects, among the most important ones). In addition, some future directions were proposed in order to valorise this troublesome plant for its nutraceutical and functional characteristics. Therefore, using *F. japonica* by exploiting its pharmacological properties through sustainable approaches such as beekeeping can lead to new and innovative opportunities, from which local communities and biodiversity can take advantage and, consequently, may reduce its negative impact. However, it should be noted that the management, control and eradication of *F. japonica* has to take into consideration the possible effects that the use of some insecticidal methods can have on the pollinators found in the invaded locations. Still, further research is needed to support the promising potential of this invasive plant as a honey source and to assess its health-supporting properties.

## Figures and Tables

**Table 1:** Phyto-chemical constituents of Japanese knotweed and identification/quantification methods.

Root extract of *R. japonica* China (*R.j.*C); *R. japonica* Poland (*R.j.*P); *R*. x *bohemica* (*R*x*b*) *R. sachalinensis* (*R.s.*)	HPLC-DAD- HR-MS gradient mode	water-formic acid (100:0.1 *v*/*v*) (solvent A) acetonitrile-formic acid (100:0.1 *v*/*v*) (solvent B)	- piceid (14.83 mg/g *R.j.*C; 7.45 mg/g *R.j.*P; 4.67 mg/g *R*x*b*) - resveratrol (1.29 mg/g *R.j.*C; 0.65 mg/g *R.j.*P; 0.52 mg/g *R*x*b*) - vanicoside B (0.64 mg/g *R.j.*C; 0.49 mg/g *R.j.*P; 0.77 mg/g *R*x*b*; 2.25 mg/g *R.s.*) - vanicoside A (0.08 mg/g *R.j.*C; 0.04 mg/g *R.j.*P; 0.12 mg/g *R*x*b*; 0.55 mg/g *R.s.*) - emodin (4.93 mg/g *R.j.*C; 4.01 mg/g *R.j.*P; 1.93 mg/g *R*x*b*; 0.13 mg/g *R.s.*) - physcion (2.96 mg/g *R.j.*C; 1.23 mg/g *R.j.*P; 1.86 mg/g *R*x*b*; 0.19 mg/g *R.s.*)	[57]
Root, stalk and leaves extract of *F. japonica* (Houtt) (*F.j.*) and *F. sachalinensis* (F. Schmidt)(*F.s.*)	LC-MS-Q/TOF isocratic mode UPLC-PDA gradient mode	methanol-acetonitrile (15:85 *v*/*v*) 0.25% aqueous acetic acid (solvent A) acetonitrile (solvent B)	- flavan-3-ols (leaves: 0.04–0.26 g/100 g *F.j.*; 0.04–0.23 g/100 g *F.s.*; stalk: 0.01–0.18 g/100 g *F.j.*; 0.02–0.24 g/100 g *F.s.*; roots: 0.02–1.48 g/100 g *F.j.*; 0.07–0.62 g/100 g *F.s.*) - phenolic acids (leaves: 0.01–0.49 g/100 g *F.j.*; 0.01–0.58 g/100 g *F.s.*; stalk: 0.01–0.08 g/100 g *F.j.*; 0.01–0.07 g/100 g *F.s.*; roots: 0.0–0.01 g/100 g *F.j.*; 0.0–0.03 g/100 g *F.s.*) - flavones/flavonols: (leaves: 0.01–1.71 g/100 g *F.j.*; 0.01–0.89 g/100 g *F.s.*; stalk: 0.01–0.28 g/100 g *F.j.*; 0.01–0.16 g/100 g *F.s.*; roots: 0.01 g/100 g *F.j.*; 0.01 g/100 g *F.s.*) - stilbenes: (leaves: 0.01–0.03 g/100 g *F.j.*; 0.01–0.02 g/100 g *F.s.*; stalk: 0.01–0.04 g/100 g *F.j.*; 0.01–0.06 g/100 g *F.s.*; roots: 0.02–0.50 g/100 g *F.j.*; 0.02–0.22 g/100 g *F.s.*)	[58]
Rhizome extracts of *R. japonica*, *R. sachalinensis* and *R.* x *bohemica*	LC-ESI-MS/MS gradient mode	water-formic acid (99.9:0.1) (solvent A) acetonitrile-formic acid (99.9:0.1) (solvent B)	- procyanidins with high degree of polymerization; dianthrone glycosides; phenylpropanoid disaccharide esters; hydroxycinnamic acid derivatives; lignin oligomers; izovitexin; izovitexin diglucoside	[63]
Leaves extract of *F. japonica* (*F.j.*) and *F.* x *bohemica* (*F*x*b*)	HPTLC–MS/MS	developing agent: 0.1% TBHQ in methanol: acetone (1:1, *v/v*)	- violaxanthin: green leaves (4.9–53.3 mg/100 g *F.j.*; 3.9–39.9 mg/100 g *F*x*b*), yellow leaves (<LOQ *F.j.*; 1.5 mg/100 g *F*x*b*), green-yellowish leaves (4.2 mg/100 g *F.j.*; 7.1–96.8 mg/100 g *F*x*b*) - neoxanthin: green leaves (38.2 mg/100 g *F.j.*; 24.4 mg/100 g *F*x*b*), yellow leaves (<LOQ *F.j.* and *F*x*b*), green-yellowish leaves (3.3 mg/100 g *F.j.*; 44.3 mg/100 g *F*x*b*) - luteoxanthin: green leaves (2.9–6.3 mg/100 g *F.j.*; 2.2–5.4 mg/100 g *F*x*b*), yellow leaves (<LOQ *F.j.* and *F*x*b*), green-yellowish leaves (1.1 mg/100 g *F.j.*; <LOQ *F*x*b*) - antheraxanthin: green leaves (10.3 mg/100 g *F.j.*; 12.8 mg/100 g *F*x*b*), yellow leaves (1.0 mg/100 g *F.j.*; 2.0 mg/100 g *F*x*b*), green-yellowish leaves (6.4 mg/100 g *F.j.*; 3.6 mg/100 g *F*x*b*) - all-trans-lutein: green leaves (144.3 mg/100 g *F.j.*; 97.1 mg/100 g *F*x*b*), yellow leaves (9.4 mg/100 g *F.j.*; 28.6 mg/100 g *F*x*b*), green-yellowish leaves (55.8 mg/100 g *F.j.*; 127.9 mg/100 g *F*x*b*) - all-trans-zeaxanthin: green leaves (3.4 mg/100 g *F.j.*; 2.7 mg/100 g *F*x*b*), yellow leaves (1.8 mg/100 g *F.j.*; 6.1 mg/100 g *F*x*b*), green-yellowish leaves (5.1 mg/100 g *F.j.*; <LOQ *F*x*b*) - 13-cis-β-carotene: green leaves (1.4 mg/100 g *F.j.*; 0.9 mg/100 g *F*x*b*), yellow leaves (<LOQ *F.j.* and *F*x*b*), green-yellowish leaves (<LOQ *F.j.*; 1.9 mg/100 g *F*x*b*) - all-trans-β-carotene: green leaves (97.3 mg/100 g *F.j.*; 68.7 mg/100 g *F*x*b*), yellow leaves (8.0 mg/100 g *F.j.*; 12.7 mg/100 g *F*x*b*), green-yellowish leaves (23.2 mg/100 g *F.j.*; 97.4 mg/100 g *F*x*b*) - 9-cis-β-carotene: green leaves (8.6 mg/100 g *F.j.*; 6.1 mg/100 g *F*x*b*), yellow leaves (<LOQ *F.j.* and *F*x*b*), green-yellowish leaves (<LOQ *F.j.*; 9.8 mg/100 g *F*x*b*) - other 8 carotenoid esters	[80]
Root, Stem, Leaf and Flower extract of *F. japonaica* (*F.j.*) and *F* x *bohemica* (*F*x*b*)	UPLC gradient mode	methanol (solvent A) water (solvent B)	- polydatin (root: 5.72–13.38 mg/g *F.j.*; 0.43–13.68 mg/g *F*x*b*; stem: 0.08–0.11 mg/g *F.j.*; 0.01–0.1 mg/g *F*x*b*; stem and leaf: 0.16–0.3 mg/g *F.j.*; 0.2–0.28 mg/g *F*x*b*; leaf: 0.13–0.25 mg/g *F.j.*; 0.05–0.41 mg/g *F*x*b*; flowers: ND) - resveratrol (root: 0.83–12.07 mg/g *F.j.*; 0.05–2.74 mg/g *F*x*b*; stem: ND; stem and leaf: 0.03–0.15 mg/g *F.j.*; 0.03–0.05 mg/g *F*x*b*; leaf: ND; flowers: ND) - emodin (root: 0.55–13.38 mg/g *F.j.*; 0.0–5.42 mg/g *F*x*b*; stem: 0.0–0.06 mg/g *F.j.*; 0.0–0.05 mg/g *F*x*b*; stem and leaf: 0.06–0.41 mg/g *F.j.*; 0.11–0.12 mg/g *F*x*b*; leaf: 0.0–0.05 mg/g *F.j.*; 0.0–0.05 mg/g *F*x*b*; flowers: ND) - physcion (root: 3.97–15.72 mg/g *F.j.*; 0.0–9.71 mg/g *F*x*b*; stem: 0.0–0.33 mg/g *F.j.*; 0.0–0.07 mg/g *F*x*b*; stem and leaf: 0.16–0.77 mg/g *F.j.*; 0.24–0.39 mg/g *F*x*b*; leaf: 0.0–0.89 mg/g *F.j.*; 0.0–0.06 mg/g *F*x*b*; flowers: ND)	[81]
Root extract of *P. cuspidatum* Sieb. et Zucc.	HSCCC gradient mode	light petroleum-ethyl acetate-water (1:5:5, *v*/*v*) light petroleum-ethyl acetate-methanol-water (3:5:4:6, *v*/*v*) light petroleum-ethyl acetate-methanol-water (3:5:7:3, *v*/*v*)	- piceid (19.3 mg), anthraglycoside B (17.6 mg) from 200 mg sample - resveratrol (18.5 mg), emodin (35.3 mg) and physcion (8.2 mg) from 220 mg sample	[84]
Sprout extract of *R. japonica* (*R.j.*) *R. sachalinensis* (*R.s.*) and *Bohemian knotweed* (*B.k.*)	HPLC-DAD gradient mode	water-acetonitrile-orthophosphoric acid (94.9:5:0.1 *v/v*)(solvent A) water-acetonitrile-orthophosphoric acid (80:19.9:0.1 *v/v*)(solvent B)	- catechin (103 mg/kg *R.j.*; 167 mg/kg *R.s.*; 42 mg/kg *B.k.*) - epicatechin (568 mg/kg *R.j.*; 674 mg/kg *R.s.*; 230 mg/kg *B.k.*) - resveratroloside (48 mg/kg *R.j.*; 31 mg/kg *R.s.*; 11 mg/kg *B.k.*) - piceid (683 mg/kg *R.j.*; 502 mg/kg *R.s.*; 215 mg.kg *B.k.*) - resveratrol (64 mg/kg *R.j.*; 29 mg/kg *R.s.*; 23 mg/kg *B.k.*)	[85]
Root extract of *P. cuspidatum* Sieb. et Zucc.	HPLC-DAD and HPLC-ESI/MS gradient mode	water:acetic acid (95.5:0.5)(solvent A) acetonitrile (solvent B)	- piceid (1.75–5.03 mg/g) - resveratrol (0.378–1.15 mg/g) - emodin-8-β-D-glucoside (3.69–9.60 mg/g) - physcion-8-β-D-glucoside (0.299–0.854 mg/g) - aloe-emodin (0.032–0.109 mg/g) - emodin (1.05–2.50 mg/g) - physcion (0.180–0.456 mg/g)	[86]
Leaves extract of *F. japonica* Houtt (*F.j.*), *F. sachalinensis* F. Schmidt (*F.s.*) and *F.* x *bohemica* (*F*x*b*)	HPTLC–MS/MS	developing agent: acetonitrile	- flavan-3-ols monomers: total content 84 mg/100 g *F.j.*; 236 mg/100 g *F.s.*; 139 mg/100 g *F*x*b* - proanthocyanidin dimers: total content 99 mg/100 g *F.j.*; 206 mg/100 g *F.s.*; 140 mg/100 g *F*x*b*	[87]
Roots extracts of *R. japonica*	UHPLC-DAD- ESI-MS^n^ gradient mode	water-formic acid (99.9:0.1) (solvent A) acetonitrile-formic acid (99.9:0.1) (solvent B)	- 4 stilbene (glycosides and aglycones) total amount of different extraction methods: 55.45 mg/g plant - 8 anthranoids (glycosides and aglycones) total amount of different extraction methods: 14.91 mg/g plant	[88]

HPLC-DAD-HR-MS-High-performance thin-layer chromatography hyphenated to high-performance liquid chromatography-diode array detection-mass spectrometry; LC-MS-Q/TOF-Liquid chromatography-photodiode detector-mass spectrometry/quadrupole time of flight; UPLC-PDA-Ultra-performance liquid chromatography with photodiode array detection; LC-ESI-MS/MS-Liquid chromatography electrospray ionization mass spectrometry/mass spectrometry; HPTLC–MS/MS-High performance thin layer chromatography and mass spectrometry; UPLC-Ultra Performance liquid chromatography; HSCCC-High-speed counter-current chromatography; HPLC-DAD-High-performance liquid chromatography with diode-array detection; HPLC-ESI/MS-High-performance liquid chromatography/electro-spray ionization tandem mass spectrometry; UHPLC-DAD-Highly sensitive ultra-high-performance liquid chromatographic-diode array detection; TBHQ- tertbuthyl hydroquinone; LOQ-limit of quantification.
